# Quantification of Fibronectin 1 (FN1) splice variants, including two novel ones, and analysis of integrins as candidate FN1 receptors in bovine preimplantation embryos

**DOI:** 10.1186/1471-213X-9-1

**Published:** 2009-01-06

**Authors:** Karen Goossens, Ann Van Soom, Alex Van Zeveren, Herman Favoreel, Luc J Peelman

**Affiliations:** 1 Department of Nutrition, Genetics and Ethology, Faculty of Veterinary Medicine, Ghent University, Heidestraat 19, 9820 Merelbeke, Belgium; 2 Department of Reproduction, Obstetrics and Herd Health, Faculty of Veterinary Medicine, Ghent University, Salisburylaan 133, 9820 Merelbeke, Belgium; 3 Department of Virology, Parasitology and Immunology, Faculty of Veterinary Medicine, Ghent University, Salisburylaan 133, 9820 Merelbeke, Belgium

## Abstract

**Background:**

Fibronectin 1 (FN1), a glycoprotein component of the extracellular matrix, exerts different functions during reproductive processes such as fertilisation, gastrulation and implantation. FN1 expression has been described to increase significantly from the morula towards the early blastocyst stage, suggesting that FN1 may also be involved in early blastocyst formation. By alternative splicing at 3 defined regions, different FN1 isoforms are generated, each with a unique biological function. The analysis of the alternative FN1 splicing on the one hand and the search for candidate FN1 receptors on the other hand during early bovine embryo development may reveal more about its function during bovine preimplantation embryo development.

**Results:**

RT-qPCR quantification of the FN1 splice isoforms in oocytes, embryos, cumulus cells and adult tissue samples revealed a large variation in overall FN1 expression and in splice variant expression. Moreover, two new FN1 transcript variants were identified, the first one expressed in bovine preimplantation embryos and the second one expressed in cumulus cells.

In the search for candidate receptors for the new embryo specific FN1 isoform, RNA expression analysis identified 5 α integrin subunits (ITGA2B, ITGA3, ITGA5, ITGA8, ITGAV) and 2 β integrin subunits (ITGB1 and ITGB3) with a similar or overlapping RNA expression pattern as compared to FN1. But double immunofluorescent stainings could not confirm complete co-localisation between FN1 and one out of 3 selected integrins alpha subunits (ITGA3, ITGA5, ITGAV).

**Conclusion:**

The existence of a new FN1 transcript variant, specifically expressed in morulae and blastocysts strengthens the idea that FN1 is involved in the process of compaction and blastocyst formation. Analysis of the integrin expression could not identify the binding partner for the embryo specific FN1 transcript variant making further steps necessary for the identification of the FN1 receptor and the downstream effects of FN1-receptor binding.

## Background

Fibronectin 1 (FN1) is a large adhesive glycoprotein of the extracellular matrix composed of 2 nearly identical subunits with a variety of binding domains for cell surface and extracellular ligands. By means of these multiple interaction sites, FN1 is involved in a variety of biological processes. Its major function is the support of cell adhesion, but FN1 also plays a role in cytoskeleton organisation, cell migration, many important physiological processes such as wound healing, thrombosis and ageing [1-3], and in diseases such as cancer, atherosclerosis and arthritis [4-7].

**Figure 1:**
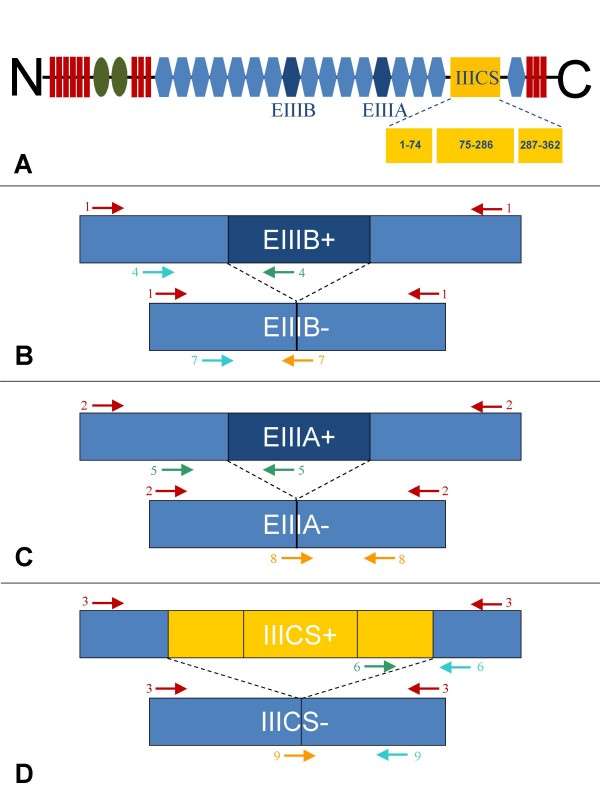
**Alternative splicing of the bovine FN1 gene**. (A) Schematic diagram of the domain structure of FN1, consisting of three repeating units, types I (red), II (green) and III (blue). Splicing can occur at two different type III repeats (EIIIB and EIIIA, dark blue) or at several locations within the IIICS region (yellow). (B) Positions of the primers for detection of the EIIIB splice variant. (C) Positions of the primers for detection of the EIIIA splice variant. (D) Positions of the primers for detection of the IIICS- and IIICS full length splice variant. Primers surrounding the alternative spliced regions (marked in red) enable the distinction between the long and short splice isoforms. The primers marked in green were used for specific amplification of the full-length isoforms and the boundary spanning primers, marked in yellow, were used for specific amplification of the short isoforms.

Structurally, FN1 is composed of 3 types of repeating structural domains (type I, II and III domains – Figure 1) and a single type III connecting segment (IIICS). This organisation is conserved among species [8]. The functional complexity of FN1 is carried out through its protein diversity, which consists of multiple isoforms including plasma FN1 [9] and cellular FN1 filaments [10]. Those FN1 isoforms are the products of a single gene, and are generated by alternative splicing at 3 sites (EIIIA, EIIIB and IIICS) of the mRNA precursor [11]. Splicing at the EIIIA and EIIIB regions happens by exon skipping, while the IIICS region can be totally included, partially included or totally excluded due to 3 alternative splice acceptor sites and one alternative splice donor site within this exon [12]. In human and bovine, about 20 different protein encoding mRNA transcripts are theoretically produced, but it is still unknown whether all of these combinations do exist *in vivo *[1,13]. The expression level of the spliced isoforms and their relative proportion changes during embryonic development and in pathological processes both spatially and temporarily [14-18]. Each alternatively spliced domain has unique biological functions and a lot of studies have been done to reveal the functions of each domain and the splicing regulation mechanisms [13,19-22]. The EIIIA and EIIIB regions are absent in plasma FN1 and EIIIA/EIIIB containing FN1 is poorly expressed in normal adult tissue but overexpressed in developing embryos, wound healing and tumours [12,23].

Integrins are heterodimeric cell surface glycoprotein receptors composed of 2 non-covalently associated subunits α and β, linking the extracellular matrix (ECM) with the intracellular cytoskeleton. Integrins mediate cell-cell and cell-matrix adhesions and the dimerisation of specific α with specific β subunits determines the specificity for the ligand as well as intracellular signalling events [24]. Upon binding of a ligand, e.g. FN1, integrins transduce signals into the cell interior (outside-in signalling), but they can also receive intracellular signals that regulate their ligand-binding affinity (inside-out signalling) [25]. At least 10 different integrins can serve as FN1 receptors. Some are specifically binding FN1 but others have multiple ligands [26].

FN1 and its receptors have different functions in mammalian reproduction and embryogenesis (sperm-oocyte interactions, implantation, and placentation) [26-30] and both the FN1 alternative splicing as well as the expression of the integrin receptors are developmentally regulated. So far, most studies have focussed on implantation and placentation, but bovine embryos are special in the way that they have a delayed implantation. In contrast to human, the bovine blastocyst elongates in the uterine lumen for several days, until attachment starts around day 19–20 post insemination (p.i.) [31,32]. Nevertheless a significant rise in bovine FN1 expression was already observed at time of transition from morula to blastocyst, more than 10 days before bovine implantation occurs. Moreover a significantly higher FN1 RNA expression was found in bovine *in vivo *golden standard blastocysts, compared to *in vitro *produced ones [33,34]. Until now, little is known about the importance of the FN1 matrix during compaction and early blastocyst formation. Takahashi *et al*. [32] described that trophectodermal cells of bovine hatched blastocyst have already acquired the ability to adhere and outgrow on FN1. But, since at this stage of development no attachment of the bovine blastocyst to the uterine epithelium is occurring, other functions for FN1 may be assumed. Moreover, a study about FN1 fibrillogenesis in Xenopus [35] suggested that the dynamic assembly of the FN1 matrix is cell-type dependent and a key event in regulating cell adhesion, migration and differentiation. Based on these data we set up the hypothesis that the embryonic FN1 matrix, formed at the onset of blastocyst formation, may have the adhesive property to link cells together and to provide a substrate for cell layer migration.

In a first step to investigate this hypothesis, and to find a link between the embryo culture conditions and alterations in FN1 expression, RT-qPCR was performed for the quantitative analysis of the FN1 splice variants in bovine oocytes, pre-implantation embryos, cumulus cells and a selection of adult tissues, followed by RT-qPCR and immunofluorescent staining for the determination of integrins as candidate FN1 receptors in bovine preimplantation embryos.

## Results

### Quantitative expression analysis of the bovine FN1 splice variants

#### Primer validation experiment

For each alternatively spliced FN1 region (EIIIB, EIIIA and IIICS), primer pairs were designed for specific amplification of both splice variants. The positions of the primers are displayed in Figure 1. Specific amplification was verified by agarose gel electrophoresis showing specific fragments of the expected sizes (Table 1), by melt curve analysis and by sequencing of the amplicons.

**Table 1:** Primer and amplicon information

**Primer**	**Target**	**Sequence**	**Amplicon size (bp)**	**Ta** **(°C)**
BtauFN1 primer pair 1	EIIIB region Standard curve	5'-CAGAGACGGGCAAGAGAGAG-3' 5'-CAGGTTGGTGAGGGTGATG-3'	+ 1060 - 787	62
BtauFN1 primer pair 2	EIIIA region Standard curve	5'-GCCCGTTTCCATCAATTACC-3' 5'-TGAGCCGGTCTGCTTGTC-3'	+ 819 - 549	62
BtauFN1 primer pair 3	IIICS region Standard curve	5'-GCCCTCAAGAACAATCAGAAG-3' 5'-TGAAATGACCACTGCCAAAG-3'	+ 808 - 448	62
BtauFN1 primer pair 4	EIIIB region Full-length region	5'-CATGCCGATCAGAGTTCCT-3' 5'-AAGAGTTTAGCGGGGTCCA-3'	202	62
BtauFN1 primer pair 5	EIIIA region Full-length region	5'-GGACCATCGAAAACGAAAAC-3' 5'-GGAATCGACATCCACATCAG-3'	195	62
BtauFN1 primer pair 6	IIICS region Full-length region	5'-AGGGGAGACGTAGACCATCA-3' 5'-GGCACTAGCAGAGGTTCCAG-3'	201	62
BtauFN1 primer pair 7	EIIIB region Boundary spanning	5'-CATGCCGATCAGAGTTCCTG-3' 5'-GAGGGACAGCTGGGATGATG-3'	130	62
BtauFN1 primer pair 8	EIIIA region Boundary spanning	5'-GGTAACCACCATTCCTGCAC-3' 5'-CCTGATACAACCACGGATGA-3'	189	62
BtauFN1 primer pair 9	IIICS region Boundary spanning	5'-GGAGGAAAAAGACAGGCCAAG-3' 5'-GGCACTAGCAGAGGTTCCAG-3'	151	62
BtauFN1 primer pair 10	Conserved region XM_864390	5'-ACTGCCCACTCCTACAACCA-3' 5'-TCTGCGAACACCACTCCA-3'	196	62
BtauITGA2B	Integrin αIIb NM_001014929	5'-TGGAAACACCCACGTCCA-3' 5'-CCAGCTTCTCCACGCTCAC-3'	155	60
BtauITGA3	Integrin α3 NM_001101900	5'-GGCAGACCTGAACAATGATG-3' 5'-CAATGCTTGCCACAGAGAAG-3'	191	61
BtauITGA4	Integrin α4 NM_174748	5'-AGACGAGGATCTCAACATCACA-3' 5'-AATGAGGCTGGACTTACAAACC-3'	147	60
BtauITGA5	Integrin α5 XM_614854	5'-GTGTGAGGCCGTGTATGAAG-3' 5'-CAGACCGAGGAGCAGGAG-3'	178	60
BtauITGA8	Integrin α8 XR_027842	5'-GCTCAAGTAGAAATAAGAGGAGT GTC-3' 5'-AGGAGGGTGTCGCTGATG-3'	167	60
BtauITGA9	Integrin α9 XM_610637	5'-ATGTGACGGGAGAGGAGGAG-3' 5'-CGAGGTTGGAGATGGAGATG-3'	243	61
BtauITGA11	Integrin α11 XM_602058	5'-CGCCAGATCACGTTTGAGA-3' 5'-ACTCCAGCACTTTGGGTGTG-3'	190	60
BtauITGAD	Integrin αD NM_001102496	5'-GACTGCTCCATCGCTGACT-3' 5'-CACGACCAATGTCTTCTTCTG-3'	141	60
BtauITGAV	Integrin αV NM_174367	5'-GGGACTTCCAGACCATCAAG-3' 5'-GTCTATATCCGTGGCTCCTTTC-3'	275	61
BtauITGB1	Integrin β1 NM_174368	5'-TGAGGTGAACAGCGAAGACA-3' 5'-TTGCACTCACACACTCGACA-3'	231	60
BtauITGB2	Integrin β2 NM_175781	5'-GGCAGAAAGCAACATCCAG-3' 5'-TGATAAGCTCCACCACGTTC-3'	137	60
BtauITGB3	Integrin β3 XM_616376	5'-TTCTCCACCAGAGGCTATCA-3' 5'-TCTGCTTCTTCACCTCCTCA-3'	131	61
BtauITGB6	Integrin β6 NM_174698	5'-ACAACGGCAATGGTTCCTTC-3' 5'-GCTCCTGCACACGCACTC-3'	299	60
BtauITGB7	Integrin β7 NM_001105365	5'-CTACAGTCGCAGCCCAGAG-3' 5'-CCGGAGGGAGAAGAGAGTG-3'	260	60

For the quantification of the amount of expression of each splice isoform, different standard curves were generated using specific PCR fragments obtained from plasmid constructs containing the specific splice variants as explained in the section Methods. After determination of the mass concentration, equimolar concentrations of the PCR products were used for the generation of ten-fold serial dilution series.

PCR efficiencies ranged from 88 to 100% and the accuracy of the standard curves was evaluated by analysing each standard dilution point as an unknown sample [36,37].

Results of the qPCR amplification of each splice variant in the presence of excess of the alternative splice variant gave equal CT values and quantities for each dilution point demonstrating the specificity of the primer pairs for each splice isoform (Additional file 1). The repeatability of the assay was good, with a standard deviation of less than 0.5 CT between technical replicates. The accuracy of the standard curves was evaluated by analysing each standard dilution point as an unknown sample [36,37]. The coefficients of variation were less than 1% for the CT values and ranged between 2 and 15% for the calculated quantities.

#### Quantification of alternative transcripts using boundary spanning primers

**Figure 2:**
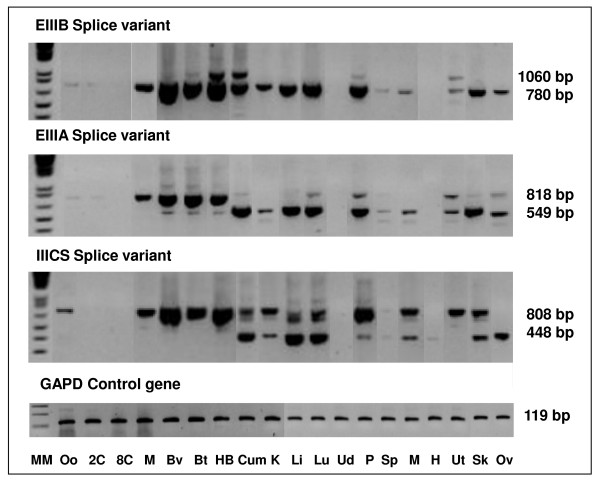
**Results of the RT-PCR on oocytes, embryos, cumulus cells and tissue samples**. The RT-PCR reactions were performed with primer pairs 1–3, surrounding the alternatively spliced regions and yielded PCR fragments of different lengths depending on the presence or absence of the alternatively splice region, and were compared to the expression of the control gene GAPD. MM: molecular marker, Oo: oocyte, 2C: *in vitro *2-cell embryo, 8C: *in vitro *8-cell embryo, M: *in vitro *morula, Bv: *in vivo *blastocyst, Bt: *in vitro *blastocyst, HB: *in vitro *hatched blastocyst, Cum: cumulus cells, K: kidney, Li: liver, Lu: Lung, Ud: udder, P: placenta, Sp: spleen, M: muscle, H: heart, Ut: uterus, Sk: skin, Ov: ovary.

In a first step, conventional RT-PCR with primers surrounding the alternatively spliced regions (Figure 1 – Table 1; Primers 1–3) were used to get an indication of the presence or absence of each splice isoform in bovine oocytes, embryos, cumulus cells and adult tissues. The results (Figure 2) demonstrated that the EIIIB region was nearly absent in bovine oocytes and embryos whereas the EIIIA region and the full-length IIICS region were highly present. Cumulus cells on the other hand, had a different expression pattern with lower expression of the EIIIB region and near absence of the EIIIA and IIICS regions. In adult tissue samples, the EIIIA and EIIIB regions were mostly absent and the presence of multiple IIICS fragments, suggest the expression of more than one transcript isoform. The two main IIICS isoforms were the IIICS null and the IIICS full-length isoforms while the intermediate IIICS forms were hardly expressed in the analysed samples.

**Figure 3:**
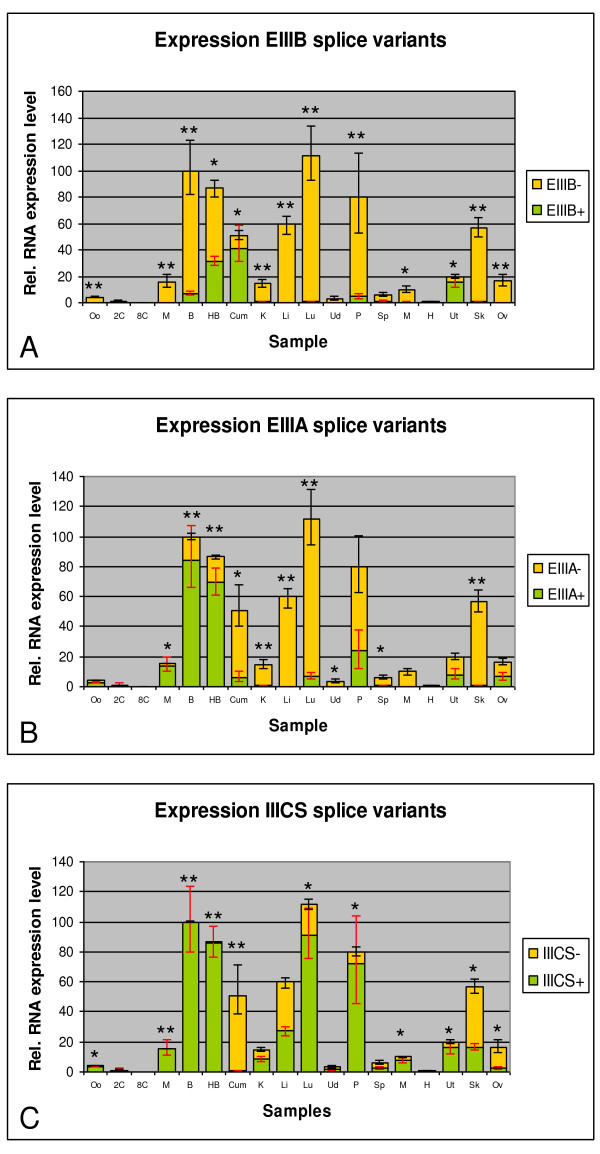
**Results of the quantitative expression analysis of the bovine FN1 splice variants**. The total FN1 RNA expression and the relative composition of each splice isoform was determined for the 3 alternatively spliced regions (A: EIIIB, B: EIIIA, C: IIICS). Oo: oocyte, 2C: *in vitro *2-cell embryo, 8C: *in vitro *8-cell embryo, M: *in vitro *morula, B: *in vitro *blastocyst, HB: *in vitro *hatched blastocyst, Cum: cumulus cells, K: kidney, Li: liver, Lu: Lung, Ud: udder, P: placenta, Sp: spleen, M: muscle, H: heart, Ut: uterus, Sk: skin, Ov: ovary. For each sample type, a t-test was performed to compared the relative expression of each splice isoform (* = P < 0.05; ** = P < 0.01).

In the second experiment, RT-qPCR with specific primers pairs was used for quantitative expression analysis of the splice isoforms in the samples (Figure 3).

The overall FN1 expression was very low in bovine oocytes and early embryonic stages and increased from the morula stage onwards, followed by a spectacular rise at the blastocyst stage (Additional file 2 for the statistical analysis). The FN1 RNA expression was variable in the adult tissue samples with very low FN1 RNA expression in udder, spleen, muscle and heart tissue, moderate expression in kidney, uterus and ovaries and high FN1 RNA expression in liver, lung, placenta and skin tissues.

The EIIIB+ isoform was absent or very low expressed in most tissue samples, except in the uterus and cumulus cells in which the EIIIB+ splice variant was predominantly expressed. During embryo development, there was a shift in the EIIIB splice isoform expression from the EIIIB- variant to the EIIIB+ variant around the hatched blastocyst stage.

The EIIIA+ isoform was the main variant in all embryonic stages. In cumulus cells and most tissue samples on the other hand, the EIIIA- variant was mainly expressed. In the reproductive tissues – placenta, uterus and ovary – the EIIIA+ variant had a higher RNA expression level compared to the other tissue samples.

The IIICS full length splice isoform was the only IIICS variant expressed during bovine preimplantation embryo development. In cumulus cells the IIIICS null isoform was virtually the only IIICS variant, whereas in most tissue samples both the null and full-length isoforms were expressed.

**Figure 4:**
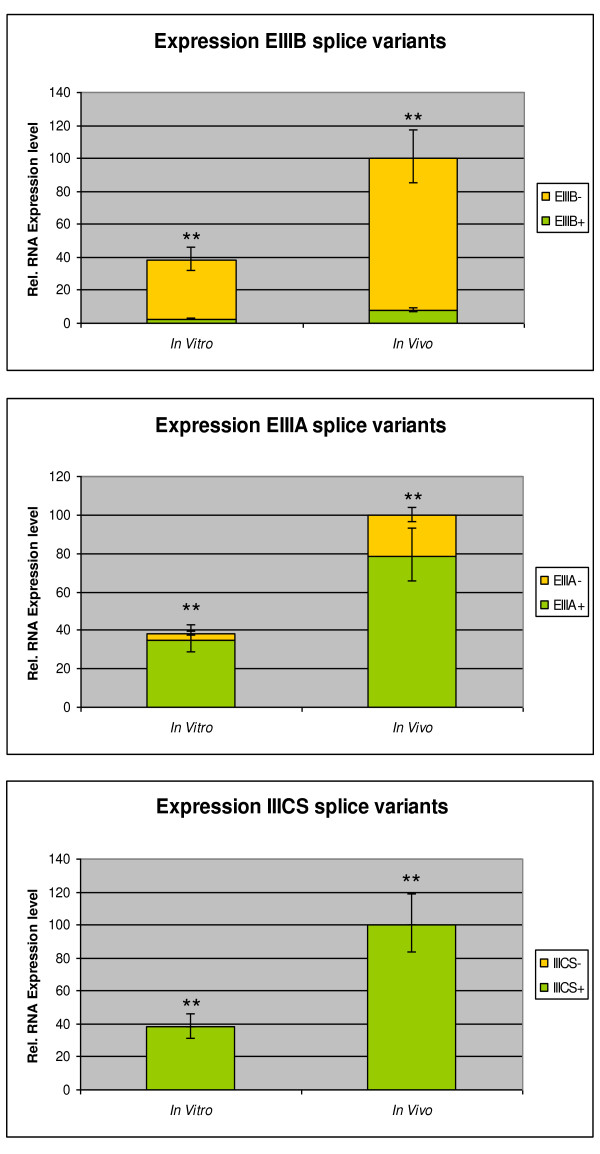
**Comparison of the FN1 isoform expression in *in vitro *versus *in vivo *produced bovine blastocysts**.

Comparison of the FN1 expression between *in vivo *and *in vitro *produced blastocysts by RT-qPCR (Figure 4) showed a 2.5 fold higher FN1 RNA expression in *in vivo *compared to *in vitro *produced blastocysts (P < 0.05). The splice variant ratios did not differ between the two groups for the EIIIB and the IIICS region (P > 0.05), and only a slight increase in EIIIA- splice variant expression was observed in *in vivo *blastocysts but this difference was not significant (P > 0.05).

When the information of the 3 regions was taken together, sequenced and compared to the 5 in silico predicted bovine FN1 splice variants in the NCBI Entrez Gene database [38] none of the described bovine splice variants agreed with the bovine embryo specific variant (EIIIB-, EIIIA+ and full-length IIICS). The embryo specific transcript variant, (in this paper nominated as FN1-ESV – Genbank Accession Number: FJ513187), expressed in both *in vivo *and *in vitro *preimplantation embryos, was similar to the in silico predicted transcript variant 12 (Accession Number: XM_874147) but had a 75 bp longer IIICS domain (amino acid sequence GHVPRGDVDHHLYPHVVGLNPNAST).

The combination of the mainly expressed subunits in cumulus cells (EIIIB+, EIIIA-, IIICS-) also yielded a new FN1 splice isoform, temporarily nominated as FN1-CSV (Genbank Accession Number: FJ513188). In tissue samples, the expression pattern was more diverse and in most of the cases a mixture of more than one transcript variant was expressed but the main transcript variant in bovine tissues agreed with the in silico predicted FN1 transcript variant 10 (Accession Number: XM_873966).

### Expression analysis of integrins as candidate FN1 receptors during bovine preimplantation embryo development

#### RNA expression analysis

Based on a literature search [24,25,27] primers were designed for 9 integrin α and 5 integrin β subunits described to act as receptors for FN1 (Table 1).

RT-qPCR analysis (Table 2 – Additional file 3) for those 14 integrins revealed that the RNA expression pattern of 6 subunits – ITGA2B, ITGA3, ITGA5, ITGA8, ITGAV and ITGB3 – followed largely the overall FN1 RNA expression during bovine preimplantation embryo development, whereas ITGA4, ITGA9 and ITGA11 were expressed in oocytes, 2-cell and 8-cell embryos but were absent in morulae and blastocysts. ITGB1 was continuously expressed during preimplantation embryo development. ITGB2 and ITGB6 on the other hand, were absent in bovine embryos and ITGAD and ITGB7 were only expressed at the hatched blastocyst stage.

**Table 2:** Overview of the integrin RNA expression, relatively compared to the FN1 RNA expression, in bovine oocytes and preimplantation embryos

Gene	Oocyte	2-cell	8-cell	Morula	Blastocyst	Hatched Blastocyst
FN1	+	-	-	+	+++	+++
**ITGA2B**	+	-	-	-	+	+++
**ITGA3**	+	+	-	-	+	+++
**ITGA5**	+	-	-	+	+++	+++
**ITGA8**	-	-	-	-	+/-	++
**ITGAV**	+	+/-	-	+	+++	+++
**ITGB3**	+	-	+/-	++	+++	++
**ITGA4**	+	+	+	-	-	-
ITGA9	+	+	+	-	-	-
ITGA11	+	+/-	+/-	-	-	-
ITGAD	-	-	-	-	-	+
ITGB1	+++	+++	++	+	+	+
ITGB2	-	-	-	-	-	-
ITGB6	-	-	-	-	-	-
ITGB7	-	-	-	-	-	+

The integrins that follow the FN1 RNA expression pattern (marked in bold) are considered as candidate receptors for the FN1-ESV isoform (-: no expression, +/-: expression in part of the samples; + low expression; ++ moderate expression, +++ high expression compared to FN1 blastocyst expression).

**Figure 5:**
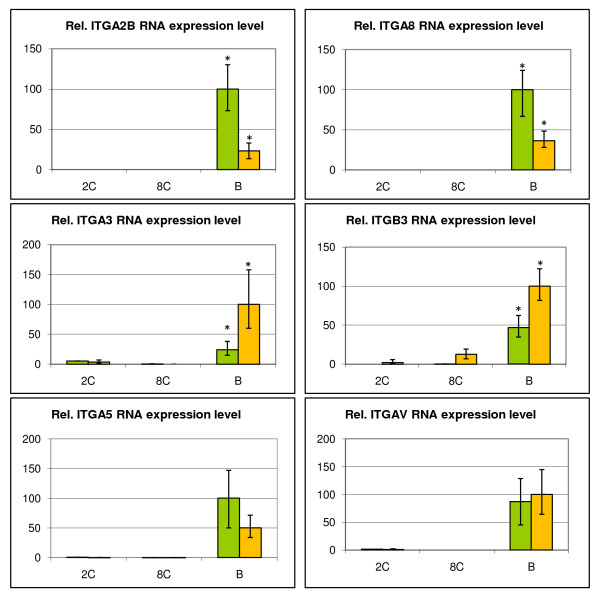
**Integrin RNA expression**. Mean relative mRNA abundance of 6 integrins at 3 different time-points during bovine preimplantation embryo development (2-cell, 8-cell and blastocyst) of *in vitro *(green) and *in vivo *(yellow) produced bovine embryos, determined by RT-qPCR. A t-test was performed and non-overlapping error bars indicate a significant difference between the according means (* = P < 0.05).

For the 6 integrin subunits that followed the FN1 RNA expression pattern (ITGA2B, ITGA3, ITGA5, ITGA8, ITGAV and ITGB3) RT-qPCR analyses were run on both *in vitro *and *in vivo *embryo samples (2-cell, 8-cell and blastocyst). The results (Figure 5) showed significant differences in RNA expression between *in vivo *and *in vitro *produced embryos for 4 of the tested integrins. ITGA2B (P = 0.0262) and ITGA8 (P = 0.0147) were significantly higher expressed in *in vitro *produced blastocyst, whereas the opposite was the case for ITGA3 (P = 0.0461) and ITGB3 (P = 0.0238). The expression of ITGB3 was not only increased at the blastocyst stage, but ITGB3 also showed a significantly higher expression in 8-cell *in vivo *embryos. The differences in RNA expression for ITGA5 and ITGAV were not significant (P > 0.05).

#### Protein expression analysis

Double immunofluorescent stainings were performed for FN1 in combination with one of the 3 integrins for which suitable antibodies were commercially available, (ITGA3, ITGA5 and ITGAV) on bovine *in vitro *produced embryos of different developmental stages (2-cell, 8-cell, morula and blastocyst; the results are not shown except for blastocysts).

**Figure 6:**
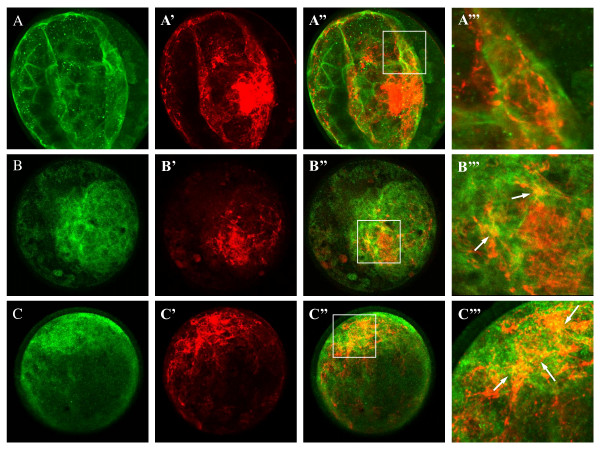
**Localisations of the FN1 and integrin subunits by double immunofluorescent staining on *in vitro *produced blastocyst**. (A) Double immunofluorescent staining for ITGA3 (A, FITC), FN1 (A', Cy5) and merged signals (A"). ITGA3 was mainly expressed at the cell membranes of the TE cells. FN1 and ITGA3 were expressed adjacent to each other but no co-localisation was found (A"'). (B) Double immunofluorescent staining for ITGA5 (B, FITC) and FN1 (B', Cy5) and merged signals (B"). (C) Double immunofluorescent staining for ITGAV (C, FITC) and FN1 (C', Cy5) and merged signals (C"). ITGA5 and ITGAV were mainly expressed in the ICM. Partial co-localisation with FN1 was observed for ITGA5 (B"') as well as ITGAV (C"') by the appearance of yellow areas when signals were merged (indicated by arrows) (Original Magnification ×400).

Bovine morulae and blastocysts were strongly positive for FN1, ITGA3, ITGA5 and ITGAV. FN1 was mainly expressed in the inner cell mass (ICM) of the blastocysts but a filamentous network of FN1 fibrils was also observed from the ICM towards the trophectodermal cells (TE) (Figure 6A', B' and 6C'). The integrin subunit ITGA3 was predominantly detected at the cell membranes of the TE whereas ITGA5 and ITGAV were both found mainly in the ICM and less in the TE (Figure 6A, B and 6C). Yellow spots on the merged images (Figure 6A", B" and 6C") showed a partial co-localization of FN1 with ITGA5 and ITGAV in the ICM and a partial co-localization between FN1 and ITGA3 in the TE.

In order to study the FN1-integrin interaction in more detail, higher magnification images (4.5× magnification; Figure 6A"', B"' and 6C"') were analysed and confirmed few yellow merged dots for FN1/ITGA5 and FN1/ITGAV double immunofluorescent stainings, indicating a slight co-localization between FN1 and those integrin subunits. However, neither for ITGA5, nor for ITGAV full colocalization with FN1 was observed.

In the TE cells, FN1 appeared as a filamentous network of fibers in close apposition to the ITGA3 positive cell membranes but true co-localization between ITGA3 and FN1 could not be observed.

## Discussion

In this study, the analysis of FN1 splice variants and integrins as candidate FN1 receptors was performed in order to test our hypothesis that bovine embryonic FN1 expressed at the morula and early blastocyst stage, may be involved in the adhesion and migration of embryonic cell layers at the onset of differentiation.

The first objective of this study was to determine the FN1 splice isoforms expressed during bovine preimplantation embryo development and to compare them with the ones expressed in oocytes, cumulus cells and adult tissue samples. The optimized RT-qPCR assay revealed large differences in overall FN1 RNA expression and, more importantly, large differences in splice variant expression.

The maternal FN1 transcript isoform, expressed in oocytes and gradually degrading in 2-cell embryos, was identical to the embryonic FN1 transcript isoform expressed from the morula stage onwards. Sequencing followed by the comparison of this splice variant (EIIIB-/EIIIA+/IIICS+) with the in silico predicted bovine FN1 transcript variants in the NCBI Entrez Gene database [38] revealed that it constitutes a new FN1 splice isoform (Genbank Accession Number: FJ513187) supporting our hypothesis that FN1 exerts a specific function during compaction and early blastocyst formation.

Previous studies [33,34] reported that *in vitro *produced embryos had a reduced FN1 RNA expression compared to *in vivo *"golden standard" embryos. *In vitro *culture conditions are largely different from the *in vivo *environment in the maternal tract and deficiency in survival factors such as cytokines and growth factors are known to have consequences for the quality of the *in vitro *embryo by induction of changes in gene expression, metabolism and even epigenetic modifications. As growth factors, cytokines, hormones and even stress are also reported to act as regulators of alternative splicing [40,41] a comparison of the FN1 splice variant expression between *in vivo *and *in vitro *blastocysts was performed. The significantly higher FN1 RNA expression found in *in vivo *blastocysts was not associated with a switch in splice variant composition. The FN1-ESV splice variant was also found in *in vivo *blastocysts and the ratios between the splice variants remained largely constant.

From the hatched blastocyst stage onwards, the expression of the EIIIB region increased, pointing to a shift in FN1 splice variant expression from the FN1-ESV isoform towards the in silico predicted FN1 isoform 2 (Accession Number: XM_864390). This shift goes together with the onset of initial differentiation of the primitive endoderm or hypoblast in cattle [42] and is not surprising since the primitive endoderm was demonstrated to be the major source of FN1 production [43]. The FN1 isoform 2 (EIIIA+/EIIIB+/IIICS+) is involved in cell lineage and tissue rearrangements [17], processes that occur extensively during early embryogenesis. The expression of a second FN1 transcript variant in hatched blastocysts supports our hypothesis that FN1 exerts multiple functions during blastocyst formation.

Another remarkable finding was the difference in FN1 isoform expression between the oocyte (and early embryos) and the surrounding cumulus cells. The cumulus cells express another new FN1 isoform, named FN1-CSV (Genbank Accession Number: FJ513188). The adhesion of spermatozoa to the oolemma is mediated by integrins recognizing the RGD sequence in glycoproteins such as FN1 and vitronectin [44]. But since a different FN1 isoform is expressed in oocytes and cumulus cells, a different function for FN1 can be expected.

In adult tissues, a variety of different splice variants was found. In general, the EIIIB and EIIIA region were excluded, agreeing with other studies describing exclusion of these regions in normal adult tissues [16,45] but inclusion during pathological conditions [4-7]. Soluble plasma FN1 (EIIIB-/EIIIA-/IIICS-) [46] is particularly secreted by hepatocytes, probably explaining the higher expression of the IIICS- variant in liver tissue. It was not the main focus of our research to study the FN1 isoform expression in adult tissue samples, but the aberrant splice variant pattern (EIIIB+/EIIIA+) in the reproductive tissues (placenta, uterus and ovary) was remarkable, although not entirely unexpected. These tissues are very proliferative and active, and the inclusion of EIIIA and EIIIB occur under such conditions [20,21,24]. Embryo implantation and placenta formation require, besides cell migration, trophoblast differentiation and villous mesenchyme formation, also intensive vascular proliferation and angiogenesis [47-49], explaining the higher inclusion of the EIIIA and EIIIB regions.

Taking a closer look at the FN1-ESV isoform, it was seen that the main difference with the most closely related isoform (in silico predicted isoform 12) was the inclusion of a 75 bp fragment containing a second RGD motif, a TYR fosforylation site and an ASN glycosylation site. The RGD motif is the recognition site for interaction with integrins and the conserved RGD motif is located in the 10^th ^type III domain, next to the PHSRN synergy site located in the 9^th ^type III domain. Upon interaction with the RGD ligand, the integrins can transduce signals into the cell interior, but they may also receive intracellular signals regulating their cell binding affinity [50]. The various α integrin subunits have different cytoplasmic tails by which they are able to activate different cellular responses upon binding to a common extracellular ligand [28]. By the binding of cytoskeleton linkage proteins such as talin, vinculin and α-actinin to their cytoplasmic tail, integrins serve as transmembrane links between extracellular contacts and the cytoskeleton [25] and act as modulators of cytoskeleton organisation, an important process during blastocyst formation. As the FN1-ESV isoform contains a second RGD motif, the study of the integrin expression in bovine embryos may help to find out more about the functions of FN1 during blastocyst formation. ITGA5B1 and ITGAVB3 are classical receptors for FN1 but at least 8 other different integrins may serve as receptors for fibronectin and many integrins can bind multiple ligands. RT-qPCR analysis was performed for 9 selected α and 5 selected β subunits. The integrin subunits following the FN1 expression profile (ITGA2B, ITGA3, ITGA5, ITGA8, ITGAV and ITGB3) and the ITGB1 subunit expressed in all the tested developmental stages, were considered as potential receptors for the new embryonic FN1 splice isoform. ITGAD and ITGB7 were only expressed at the hatched blastocyst stage, making them good candidate receptors for FN1 isoform 2. Furthermore, remarkable differences in integrin RNA expression were found between *in vivo *and *in vitro *produced blastocysts. The embryonic origin might not only influence the FN1 expression but also the integrin expression, or ligand and receptor might regulate each others expression.

The double immunofluorescent stainings on *in vitro *produced bovine blastocysts found ITGA3 mainly located in the TE cells, whereas ITGA5 and ITGAV were stronger expressed in the ICM. Although the integrins were expressed in close proximity to FN1, there was no full co-localisation between FN1 and one of 3 the receptors. Either, none of the tested integrins was the unique FN1 receptor, each integrin receptor can bind to other ligands besides FN1, or the FN1-ESV isoform can interact with more than one integrin receptor by the dual RGD sequences. One should keep in mind that analogous to the differences observed for RNA expression, differences in protein expression can exist between *in vitro *and *in vivo *golden standard embryos. ITGA2B and ITGA8 were not tested in this study due to the lack of suitable, specific antibodies. Our approach using RNA expression pattern analysis and double immunofluorescent stainings excluded some integrins as candidate binding partners for the embryo specific FN1 isoform, but we were not able to identify one specific FN1 receptor in bovine blastocysts. The application of other molecular techniques such as the yeast two hybrid method [51] or the MAPPIT strategy [52] will be necessary for further analysis of the FN1-integrin interaction in bovine blastocysts in order to find out more about the function of FN1 during early blastocyst formation.

## Conclusion

In this study, we have established a reliable assay for mRNA quantification of different FN1 splice isoforms. The assay was used for FN1 expression analysis in bovine oocytes, embryos, cumulus cells and adult tissues and as such we have identified 2 novel FN1 isoforms, specifically expressed in early embryos and cumulus cells, respectively. The existence of the FN1-ESV transcript variant strengthens our hypothesis that FN1 may exert a specific function during blastocyst formation.

Expression analysis of integrins, the main family of FN1 receptors, during bovine preimplantation embryo development was a first step towards unravelling the FN1 function during blastocyst formation. RT-PCR identified candidate binding partners for the FN1-ESV isoform, but detailed identification of its receptor needs further investigation to test our hypothesis that the embryonic FN1 matrix has the adhesive property to link cells together and provides a substrate for cell layer migration.

## Methods

### Sample collection

#### In vivo embryo production

*In vivo *embryos were produced according to the protocol described in Goossens *et al*. [33]. This protocol has been approved by the local ethical committee and was done after superovulation and artificial insemination of 4 Holstein cows for the collection of 2-cell and 8-cell embryos and 2 Holstein heifers for the collection of blastocysts. The same bull was used for *in vivo *and *in vitro *insemination. Four 2-cell and four 8-cell embryos were recovered from the reproductive tract of slaughtered donor animals, on 36 hours respectively 84 hours after artificial insemination (AI). The embryos were recovered from the oviducts by flushing with warm PBS supplemented with 2% FCS. Four blastocysts were recovered by nonsurgical uterine flushing with 0.5 l of PBS on day 7 after AI.

#### In vitro embryo production

Bovine embryos were produced by routine *in vitro *methods as described by Vandaele et al. [53]. Briefly, bovine cumulus-oocyte complexes (COCs) were aspirated from ovaries collected at a local slaughterhouse. Immature COCs were recovered from follicular fluid, washed two times in HEPES-TALP and matured for 20 to 24 hrs in groups of 100 in 500 μl modified bicarbonate buffered TCM199 (Gibco BRL, Belgium) supplemented with 20% heat-inactivated FCS (Sigma-Aldrich, Belgium) for 20–24 hrs at 38.5°C in a humidified 5% CO_2 _incubator.

Frozen-thawed bovine sperm was separated over a Percoll gradient (45% and 90%; Pharmacia, Sweden), washed and diluted in IVF-TALP consisting of bicarbonate buffered Tyrode solution, supplemented with BSA (6 mg/ml) and heparin (25 μg/ml) to a final sperm concentration of 1 × 10^6^spermatozoa/ml. The matured COCs were washed in 500 μl IVF-TALP and incubated with sperm. After 20 hrs co-incubation, the presumed zygotes were vortexed to remove excess sperm and cumulus cells. The complete removal of the cumulus cells was checked by microscopy. The zygotes were washed and placed in groups of 25 in 50 μl droplets of synthetic oviduct fluid supplemented with 5% foetal calf serum and cultured up to the desired stages (2-cell, 8-cell, morula, blastocyst and hatched blastocyst) at 38.5°C in 5% CO_2_, 5% O_2 _and 90% N_2_.

#### Cumulus cell culture

Matured oocytes were vortexed in 500 μl IVF-TALP to remove the surrounding cumulus cells. The oocytes were removed and the remaining medium containing the cumulus cells was centrifuged for 10 min at 750 × g. After centrifugation, the pellet of cumulus cells was suspended in cumulus medium containing TCM-199 (Gibco BRL, Belgium), Gentamycin and 10% FCS (Sigma-Aldrich, Belgium). The cumulus cells were cultured for 7 days in 4-well plates, preincubated with 300 μl of FCS to enhance cell attachment, in a humidified 5% CO_2 _incubator. The cumulus medium was renewed every 2 to 3 days with freshly prepared cumulus medium.

After 7 days, the monolayer of cumulus cells was scraped from the bottom of the 4-well plate, collected in a microcentrifugation tube, washed 3 times with PBS and immediately frozen at -80°C until RNA extraction.

#### Collection of bovine tissues

In a commercial slaughterhouse, different bovine tissue samples (kidney, liver, lung, udder, placenta, spleen, muscle, heart, uterus, skin and ovary) were carefully collected from 4 different Holstein cows. The samples were immediately submerged in RNAlater (Sigma-Aldrich, Belgium) for RNA preservation, after which they were crushed to powder with liquid nitrogen, subdivided per 80–100 mg and stored at -80°C, until total RNA extraction.

### RNA extraction and cDNA synthesis

Total RNA was isolated from single oocytes (6×), single embryos (6× 2-cell, 6× 8-cell, 6× morulae, 6× blastocysts and 4× hatched blastocysts for the *in vitro *embryos; 4× 2-cell, 4× 8-cell and 4× blastocysts for the *in vivo *embryos) and the pellets of cumulus cells (4×) using the PicoPure RNA Isolation Kit (Arcturus, CA) according to the manufacturer's instructions. An in-solution DNase digestion was carried out to remove any contaminating DNA by treating the total RNA with 2 units of RQ1 DNase (Promega, The Netherlands) during 30 min, followed by a spin-column purification (Microcon YM-100, Millipore, Belgium).

Total RNA was extracted from the bovine tissue samples (4 samples/tissue) using the Aurum Total RNA Fatty and Fibrous Tissue Kit (Bio-Rad, Belgium) according to the products manual. An on-column DNase I digest was performed during the RNA extraction protocol.

A minus RT control was performed with primers for GAPD to check the removal of the genomic DNA and First-strand cDNA was synthesized from the total amount of RNA using the iScript cDNA synthesis kit (Bio-Rad, Belgium) following the manufacturer's instructions. The iScript Reverse Transcriptase is a modified MMLV-derived reverse transcriptase and the iScript Reaction Mix contains both oligo(dT) and random primers. After the RT reaction and RT control with primers for GAPD, the cDNA was 2.5 times diluted in 10 mM Tris HCl pH 8.0.

### Primer design

All the primers were designed using the Primer 3 software [54] and are based on the sequences available in NCBI GenBank [38] (Table 1).

For each region (EIIIB, EIIIA and IIICS) 3 sets of primers were designed. Primer pairs 1–3 (Figure 1; Table 1) are surrounding the alternative spliced regions and make the distinction between the presence and the absence of the alternatively spliced regions possible. These primers were also used for the generation of the specific standards. Primer pairs 4–6 (Figure 1; Table 1) are used for specific amplification of the full-length splice regions (EIIIB+, EIIIA+ and IIICS+) with one of the primers located in the specific spliced region. Primer pairs 7–9 (Figure 1; Table 1) are boundary spanning primers used for specific amplification of the EIIIB-, EIIIA- and IIICS- regions.

Additionally, primers were designed for the RNA expression analysis of 9 different integrin α subunits and 5 different integrin β subunits (Table 1), known from literature to be candidate receptors for FN1.

All the PCR amplicons were sequenced for verification.

### Reverse Transcription qPCR

#### Generation of splice variant specific qPCR standards

The FN1 regions spanning EIIIB, EIIIA and IIICS were amplified with primer pairs 1 to 3 respectively, using pooled cDNA from bovine oocytes, embryos, cumulus cells and adult tissues. The generated PCR products (Table 1) were cloned into a pCR 2.1 vector (Invitrogen, Belgium) and transformed into competent DH5α E. coli cells (Invitrogen, Belgium). Clones were randomly picked and, after sequencing of the inserts, used for generation of gene-specific qPCR standards.

The cDNA fragment of each isoform was amplified by using 20 ng of the specific plasmids as the template for a PCR reaction. PCR fragments were run on a 2% agarose gel, excised and eluted using the Geneclean II kit (Q-Bio gene – MP Biomedicals, Belgium). PCR fragments were quantified using the PicoGreen Kit (Molecular Probes – Invitrogen, Belgium) on a NanoDrop 3300 Fluorospectrometer (Isogen Life Science B. V., Belgium) and the molar concentration of each PCR product was calculated on the basis of the mass concentration and the length in base pairs of each fragment as described in Vandenbroucke et al. [36]. Standard curves are generated by 10-fold serial dilutions of specific PCR products as validated by Vandenbroucke et al. [36].

#### Quantification of alternative transcripts using boundary spanning primers

Primer pairs designed for specific detection of each splice variant (Table 1, primers 4–9) were used in combination with SYBR Green I (Platinum SYBR Green qPCR SuperMix-UDG; Invitrogen, Belgium).

First, the specificity of the primers was tested by amplifying each splice variant in the presence of excess on the alternative splice form. In concrete terms this means that for each alternatively spliced region, a dilution series of the PCR product of the short transcript was mixed with excess of the PCR product of the full-length transcript or with equal amounts of water in a second and similar series of dilution. The same was done for each of the full-length transcripts.

Each qPCR reaction contained 1× SYBR Green I PCR Mix (Platinum SYBR Green qPCR SuperMix-UDG; Invitrogen, Belgium), 200 nM of each specific primer and 2.5 μl of sample. After a 2 min UDG incubation step at 50°C and an initial denaturation step at 95°C for 3 min to activate the *Taq *DNA polymerase, the PCR programme consisted of 45 cycles of denaturation at 95°C for 20 sec and a combined primer annealing/extension at the specific and optimised annealing temperature (Table 1) for 40 sec during which fluorescence was measured. A melt curve was produced to confirm a single gene-specific peak and to detect primer/dimer formation by heating the samples from 70 to 95°C in 0.5°C increments with a dwell time at each temperature of 10 sec while continuously monitoring the fluorescence. PCR efficiencies were calculated using a relative standard curve derived from each specific PCR product (a ten-fold dilution series with five measuring points). For each reaction two technical replicates were run, whereby a no-template control was included.

The geometric mean of three validated reference genes (YWHAZ, GAPD and SDHA) [55] was used to calculate an accurate normalisation factor for normalisation of the total FN1 RNA expression level. The mean quantity of each transcript (raw data) was divided by the respective normalisation factor to obtain a normalised value for each transcript. The relative proportion of each splice variant was determined by interpolating the CT values of the unknown samples to the corresponding standard curve and subsequently used for the calculation of the composition of the total FN1 expression.

#### Expression analysis of integrins in bovine preimplantation embryos

In a first screening experiment, the expression profiles of the 14 selected integrin subunits (Table 2) were determined in different stages of embryonic development using RT-qPCR as previously described. In a second experiment the integrin expression was compared between *in vivo *and *in vitro *produced embryos (2-cell, 8-cell and blastocysts), also by RT-qPCR.

### Statistical analysis

The gene expression levels were analysed by calculating 95% confidence intervals after logarithmic transformations, whereby non-overlapping intervals denote significant differences at the 0.05 level.

Statistical tests were performed using the GraphPad Instat program. An ANOVA test was performed to compare the overall FN1 expression between oocytes, embryos, cumulus cells and adult tissue samples. A t-test was used to compare the relative FN1 splice variant expression in each sample type. An (unpaired) t-test was used for comparison of the FN1 splice variant and the integrin expression between *in vivo *and *in vitro *produced embryos. P-values < 0.05 were considered statistically significant.

### Immunofluorescent stainings

Double immunofluorescent stainings were performed for FN1 in combination with a candidate FN1 receptor (ITGA3, ITGA5 or ITGAV). A primary mouse monoclonal antibody (AB6328 – Abcam, UK) against FN1 was used in combination with a secondary goat-anti-mouse antibody labelled with Cy5 (Chemicon – Millipore, Belgium). The primary rabbit integrin antibodies (ITGA5 (C2405-02D): USBiological – Immunosource, Belgium; ITGA3 (AB1920) and ITGAV (AB1930): Chemicon – Millipore, Belgium), guaranteed to have no-cross reactivity with other integrins were used in combination with a secondary goat-anti-rabbit antibody labeled with FITC (Molecular Probes, The Netherlands).

*In vitro *blastocysts were selected from the culture media at day 7 p.i., washed 3 times in PBS and fixed with ice cold acetone for 10 min at 4°C. After washing in polyvinyl pyrrolidone (PVP; 1 mg PVP/ml PBS), non-specific binding sites were blocked by incubating the embryos with 10% goat serum in PVP for 30 min. After 3 washes in PVP the embryos were incubated with the mouse primary, monoclonal antibody against FN1 (1/100) for 2 hours at 37°C, washed again 3 times in PVP and then incubated overnight at 4°C with the specific rabbit antibody against one of the 3 selected integrins (1/100). Afterwards, the embryos were washed 3 times with PVP, incubated with the secondary goat-anti-mouse antibody labelled with Cy5 for 1 hour at 37°C (1/100), washed again with PVP and incubated with the secondary goat-anti-rabbit antibody labelled with FITC (1/100).

The nuclei were stained with Hoechst 33258 (Molecular Probes, Belgium) for 10 min at room temperature and immediately mounted in a droplet of glycerol with (25 mg/ml) 1,4-diazabicyclo (2.2.2) octane (DABCO; Acros, Belgium) on slides with vaseline bridges.

Negative controls (by replacing the primary antibody with goat serum), double negative controls (only Hoechst staining) and controls for cross reactivity of the antibodies were performed simultaneously to check for non-specific binding and for auto-fluorescence. A monolayer of cultured cumulus cells was used as a positive control.

Samples were evaluated using a Leica DM/RBE fluorescence microscope (Leica Microsystems Belgium) and a Nikon C1 confocal laser scanning module attached to a motorized Nikon TE2000-E inverted microscope (Nikon Benelux, Belgium) equipped with a Plan Apo 40× 0.95 NA air objective and suitable optical elements to acquire differential interference contrast (DIC) transmission images. An argon 488 nm laser, a Helium-neon 633 nm laser, and a 351 nm UV-laser were used for the excitation of FITC, Cy5, and Hoechst, respectively.

## Authors' contributions

KG performed all the experimental procedures and was the primary author of the manuscript. AVS contributed to the design of the IVF experiments. HF helped with the fluorescent microscopy and the interpretation of the images. AVZ and LJP participated in the design of the project, helped to draft the manuscript and supervised the study. All authors read and approved the final manuscript.

## Supplementary Material

Additional file 1 **FN1 primer validation experiment. Results of the PCR amplification of each splice variant in the presence of excess of the alternative splice variant**. The measuring points marked in blue indicate the standards diluted in water, while the measuring points in red represents the standards diluted in excess of the alternative variant. Both series gave equal CT values and quantities for each dilution point. Click here for file

Additional file 2 **Statistical analysis of the overall FN1 expression levels between the different sample types**. An ANOVA test was used to compare the overall FN1 expression between the different sample combinations (NS: not significant P > 0.05; * = P < 0.05; ** = P < 0.01). Click here for file

Additional file 3 **RT-qPCR analysis for FN1 and Integrin subunits**. Relative FN1 and Intergin mRNA expression levels in bovine oocytes and in *vitro *produced embryos determined by RT-qPCR. For each gene, the expression levels were compared to the highest value, set at 100%. Oo: oocyte, 2C: *in vitro *2-cell, 8C: *in vitro *8-cell, M: *in vitro *morula, B: *in vitro *blastocyst, HB: *in vitro *hatched blastocyst. Click here for file
