# Supplementary material for: Quantification of Fibronectin 1 (FN1) splice variants, including two novel ones, and analysis of integrins as candidate FN1 receptors in bovine preimplantation embryos
Source: BMC Dev Biol. 2009 Jan 6;9:1. doi: 10.1186/1471-213X-9-1 (PMC2648952; doi:10.1186/1471-213X-9-1)
Supplement: Additional file 1 — FN1 primer validation experiment. Results of the PCR amplification of each splice variant in the presence of excess of the alternative splice variant. The measuring points marked in blue indicate the standards diluted in water, while the measuring points in red represents the standards diluted in excess of the alternative variant. Both series gave equal CT values and quantities for each dilution point. [file 1471-213X-9-1-S1.pdf]

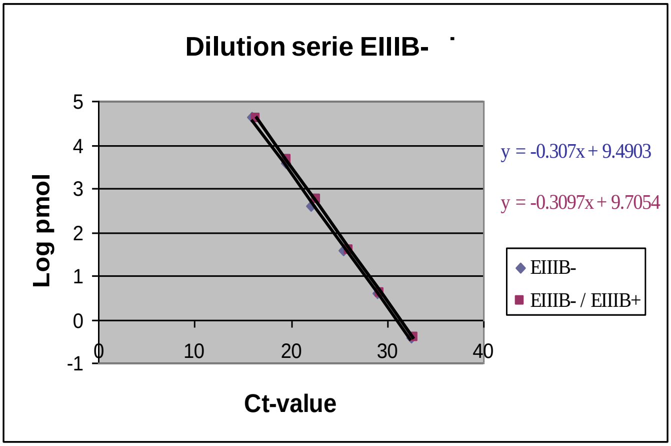
Dilution serie EIIIB-
Log pmol
Ct-value
y = -0.307x + 9.4903
y = -0.3097x + 9.7054
EIIIB-
EIIIB- / EIIIB+

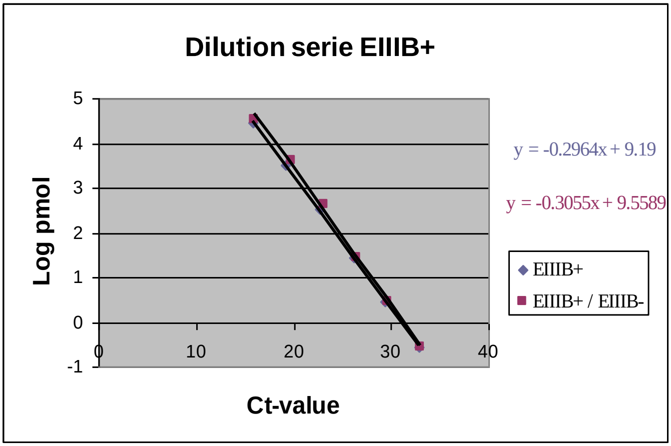
Dilution serie EIIIB+
Log pmol
Ct-value
y = -0.2964x + 9.19
y = -0.3055x + 9.5589
EIIIB+
EIIIB+ / EIIIB-

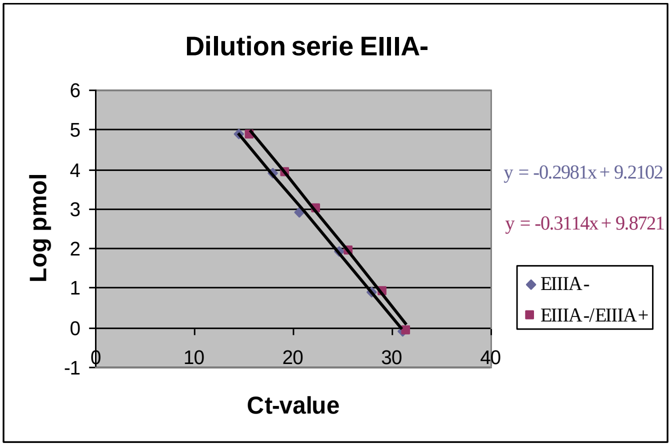
Dilution serie EIIIA-
Log pmol
Ct-value
y = -0.2981x + 9.2102
y = -0.3114x + 9.8721
EIIIA-
EIIIA-/EIIIA+

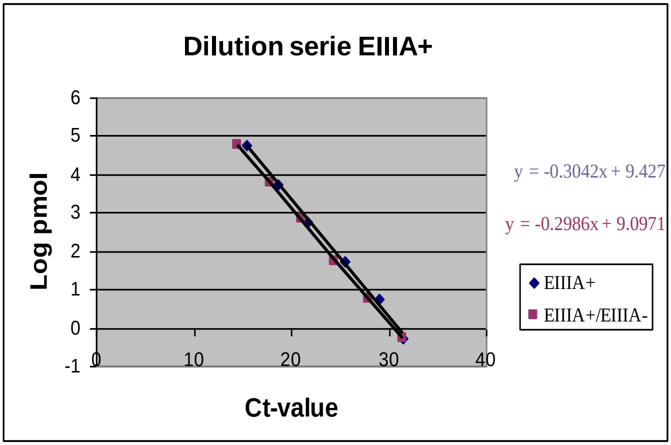
Dilution serie EIIIA+
Log pmol
Ct-value
y = -0.3042x + 9.427
y = -0.2986x + 9.0971
EIIIA+
EIIIA+/EIIIA-

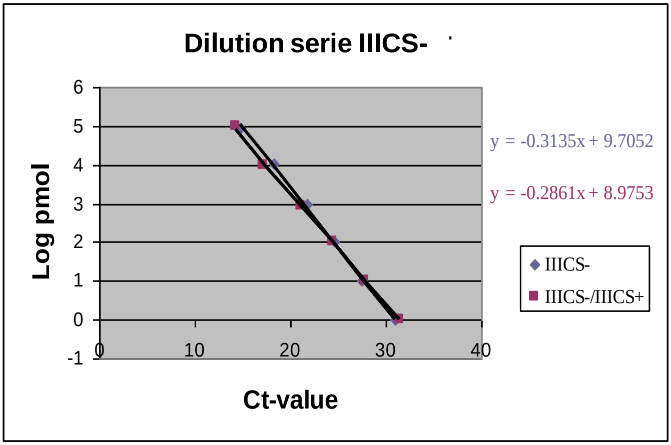
Dilution serie IIICS-
Log pmol
Ct-value
y = -0.3135x + 9.7052
y = -0.2861x + 8.9753
IIICS-
IIICS-/IIICS+

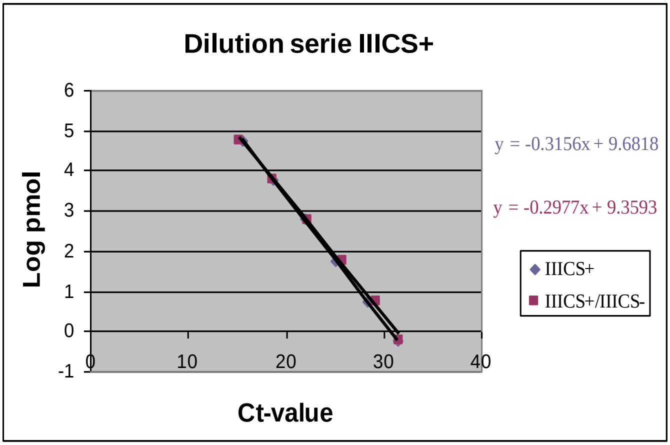
Dilution serie IIICS+
Log pmol
Ct-value
y = -0.3156x + 9.6818
y = -0.2977x + 9.3593
IIICS+
IIICS+/IIICS-
