# Supplementary figures and images for: Quantification of Fibronectin 1 (FN1) splice variants, including two novel ones, and analysis of integrins as candidate FN1 receptors in bovine preimplantation embryos
Source: BMC Dev Biol. 2009 Jan 6;9:1. doi: 10.1186/1471-213X-9-1 (PMC2648952; doi:10.1186/1471-213X-9-1)

**FN1**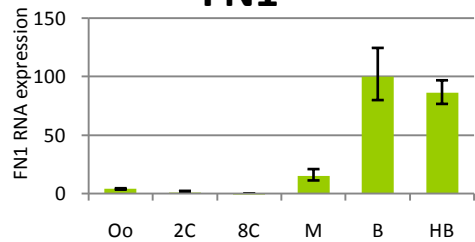**ITGA2B**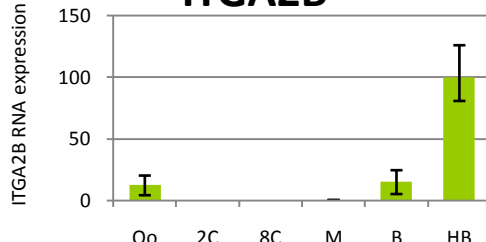**ITGA3**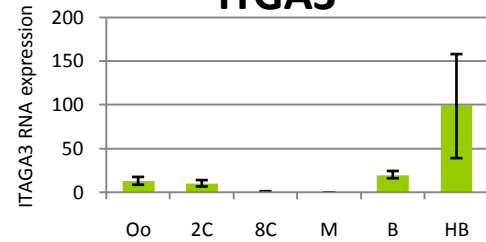**ITGA4**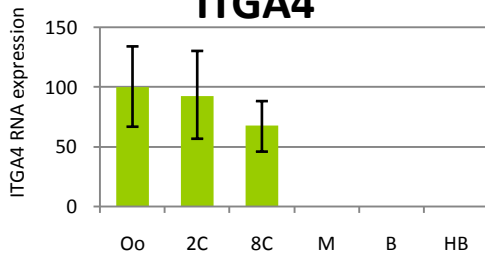**ITGA5**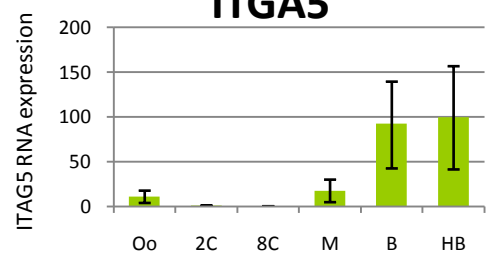**ITGA8**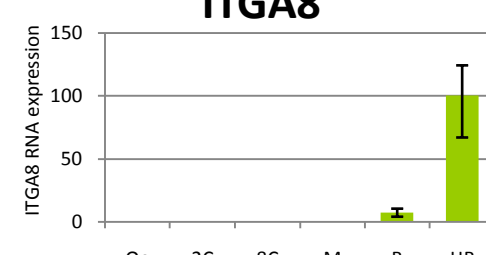**ITGA9**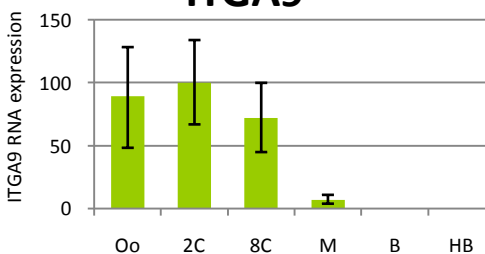**ITGA11**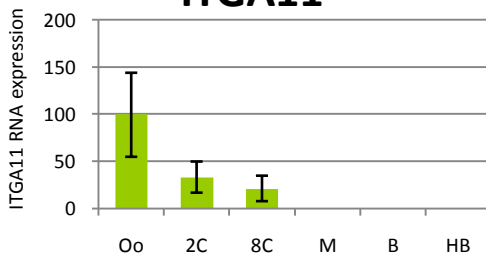**ITGAV**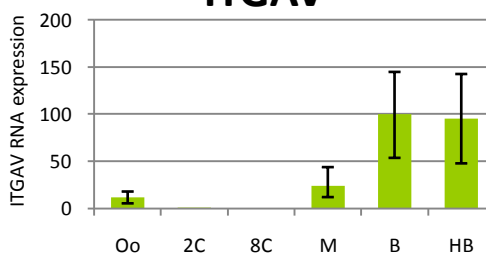**ITGB1**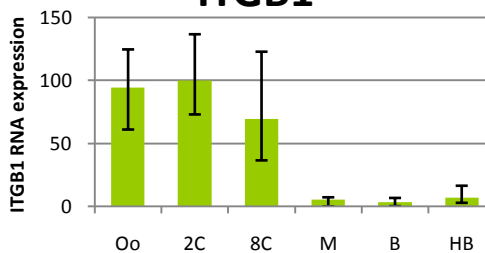**ITGB3**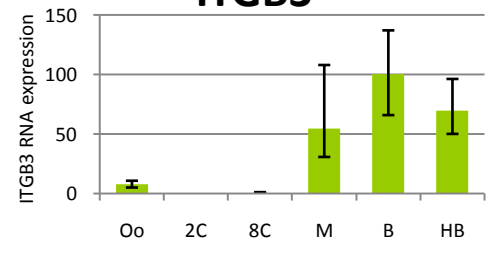

Supplement: Additional file 3 — RT-qPCR analysis for FN1 and Integrin subunits. Relative FN1 and Intergin mRNA expression levels in bovine oocytes and in vitro produced embryos determined by RT-qPCR. For each gene, the expression levels were compared to the highest value, set at 100%. Oo: oocyte, 2C: in vitro 2-cell, 8C: in vitro 8-cell, M: in vitro morula, B: in vitro blastocyst, HB: in vitro hatched blastocyst. [file 1471-213X-9-1-S3.pdf]
